# A Bayesian model of distance perception from ocular convergence

**DOI:** 10.1371/journal.pcbi.1013506

**Published:** 2025-10-03

**Authors:** Peter Scarfe, Paul B. Hibbard

**Affiliations:** 1 Vision and Haptics Laboratory, School of Psychology and Clinical Language Sciences, University of Reading, Reading, United Kingdom; 2 Department of Psychology, University of Stirling, Stirling, Scotland, United Kingdom; Utrecht University: Universiteit Utrecht, NETHERLANDS, KINGDOM OF THE

## Abstract

Ocular convergence is one of the critical cues from which to estimate the absolute distance to objects in the world, because unlike most other distance cues a one-to-one mapping exists between absolute distance and ocular convergence. However, even when accurately converging their eyes on an object, humans tend to underestimate its distance, particularly for more distant objects. This systematic bias in distance perception has yet to be explained and questions the utility of vergence as an absolute distance cue. Here we present a probabilistic geometric model that shows how distance underestimation can be explained by the visual system estimating the most likely distance in the world to have caused an accurate, but noisy, ocular convergence signal. Furthermore, we find that the noise in the vergence signal needed to account for human distance underestimation is comparable to that experimentally measured. Critically, our results depend on the formulation of a likelihood function that takes account of the generative function relating distance to ocular convergence.

## Author summary

Since at least the time of Descartes the ocular convergence state of the eyes has been considered one of the most important cues to distance in the world. However, despite accurate fixation, humans underestimate distance from ocular convergence, particularly for more distant objects. This questions the utility of one of the primary cues from which distance could be estimated. Here we present a rigorous probabilistic analysis of how humans might estimate distance from ocular convergence. This analysis shows that the experimentally observed distance underestimation can be explained by observers estimating the most likely distance in the world to have caused the accurate but uncertain measurement of the convergence state of the eyes. The level of uncertainty needed to account for observed distance underestimation is consistent with that experimentally measured.

## Introduction

### Estimating distance from ocular convergence

To interact successfully with our environment, we need to infer distal properties of the world, such as distance, depth and shape, from proximal sources of sensory information. Proximal sensory data is generally considered to consist of quasi-independent sources of information termed ‘cues’, each of which gives rise to largely statistically separable perceptual estimates of a given distal world property [1,2]. For the visual modality, this involves making inferences from both retinal and extra-retinal cues [3]. Retinal cues are available from the two-dimensional images of the world projected to the back of each eye and include cues such as perspective [4], texture [5,6], shading [7] and binocular disparity [8]. Extra-retinal cues are not contained within these images and include cues such as the physical orientation of the eyes in their orbits [9,10], and information about the movement of the eyes [11,12] and body [13] over time.

Since at least the time of Descartes, the extra-retinal cue of ocular convergence (or more succinctly vergence) has been considered a particularly important cue to absolute distance [8]. Absolute distance is defined as the Euclidean distance between the cyclopean eye and a 3D coordinate in the world. In the present paper to be succinct we will use the term “distance” interchangeably with “absolute distance”. Where needed we will draw clear distinctions, e.g., between absolute and relative distance. Vergence is defined as the horizontal angular difference between the physical orientations of the two eyes in their orbits [14] and if the eyes are converged on a point in the world, for a particular version angle, there is a geometric one-to-one mapping between vergence and the absolute distance to that point. This is absent for virtually all other visual cues [8,15–17].

**Fig 1:**
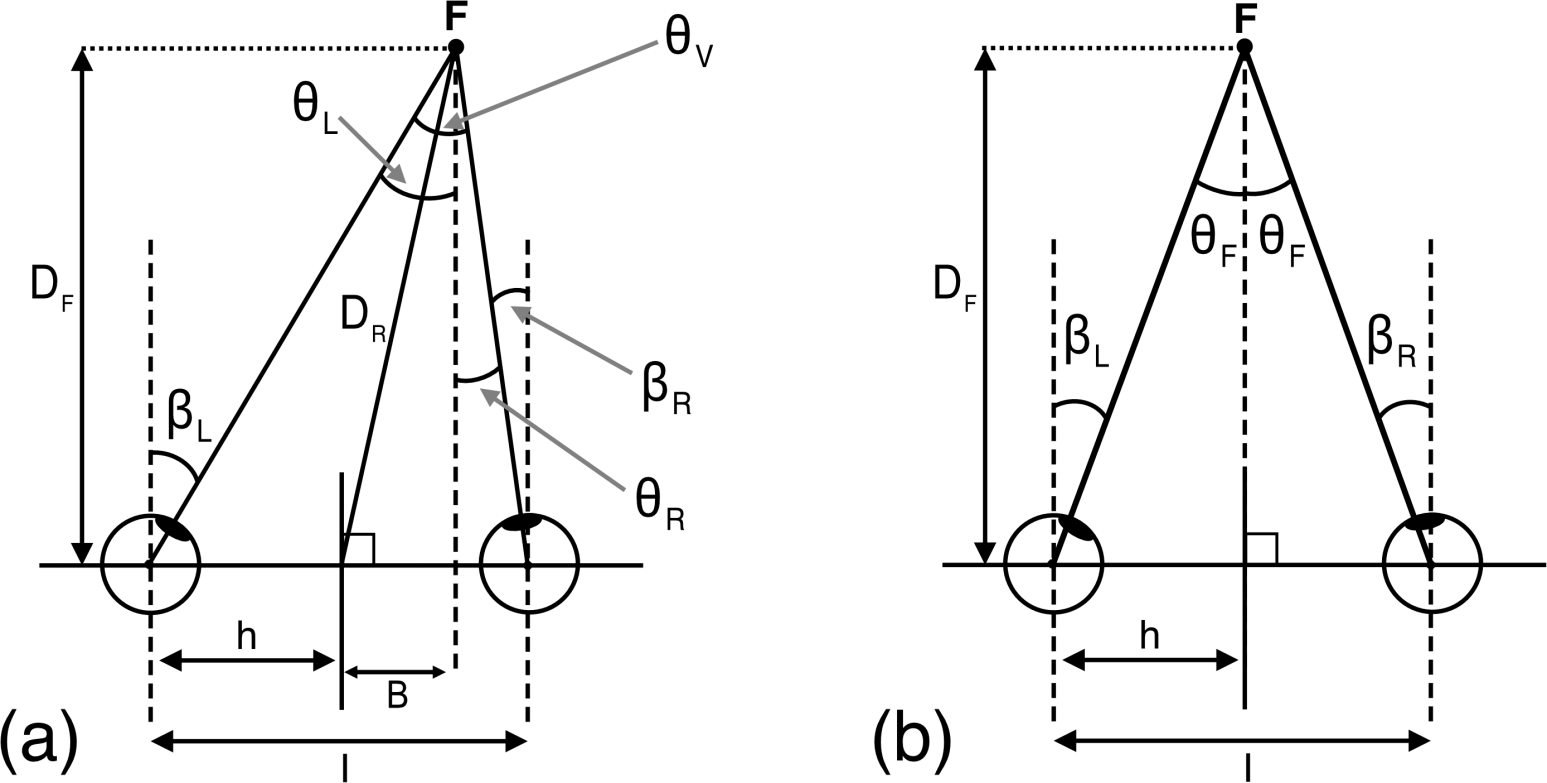
Diagram showing the geometry of estimating absolute distance from ocular convergence. (a) shows the geometry for the general case of a point of fixation off the median plane and (b) for the case in which the observer fixates a point on the median plane. See main text for details.


The geometry of vergence is shown in Fig 1a. Following standard convention, we have defined the interocular distance to be the distance between the centre of rotation of each eye and considered the eyes to be rigid, circular and to rotate about their centres (see S1 Text for discussion). With these assumptions, $D_{F}$ is the absolute distance of the fixated F and $D_{R}$ is the radial distance to that same point. I is the observer’s interocular distance and h half this value. The directions of gaze for the left and right eyes relative to straight ahead are $\beta_{L}$ and $\beta_{R}$, where $\beta_{L}=\theta_{L}$ and $\beta_{R}=\theta_{R}$, defining right angled triangles given by

$$\theta_{L}={\text{tan}}^{-\text{1}}\left(\frac{h+B}{D_{F}}\right)$$ (1)

and


$$\theta_{R}={\text{tan}}^{-\text{1}}\left(\frac{h-B}{D_{F}}\right)$$ (2)

The vergence angle, $\theta_{V}$, is given by


$${\theta_{V}=\theta }_{L}+\theta_{R}$$ (3)

Distance estimation from vergence has primarily been studied experimentally when observers are estimating the distance of objects placed directly in front of them on either (a) the median plane (Fig 1b) [10] or (b) directly in front of one eye [9]. Under both circumstances the geometry of vergence is simplified to a right-angled triangle with a base of length h or I respectively. In the case of a point on the median plane, $D_{F}=D_{R}$ and B=0, and the half vergence angle for each eye, $\theta_{F}$, is given by


$$\theta_{F}={\text{tan}}^{-\text{1}}\left(\frac{h}{D_{F}}\right)$$ (4)

Equation 4 defines the mathematical relationship relating the distal property of distance in the world ($D_{F}$) to the measured proximal vergence signal ($\theta_{F}$). By rearranging to make $D_{F}$ the subject, one can see that estimation of distance from vergence requires knowledge of the convergence state of the eyes and the interocular separation


$$D_{F}=\frac{h}{\text{tan}\left(\theta_{F}\right)}$$ (5)

**Fig 2:**
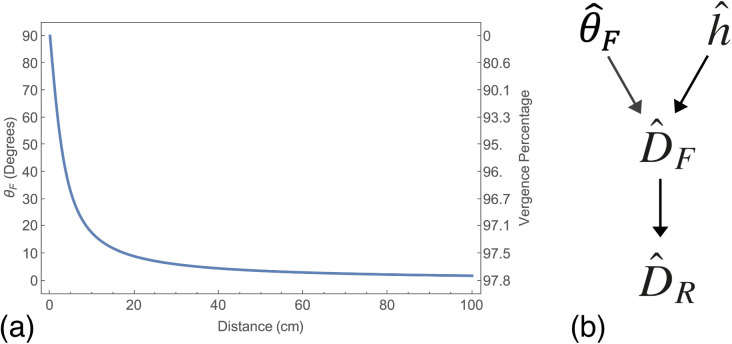
Vergence as a cue to absolute distance. (a) Plot shows the magnitude of the half vergence signal $\theta_{F}$ over distance for a person with a 6.5 cm interocular distance. This is also plotted as a percentage of the total vergence range. (b) Shows the steps in estimating distance from vergence. ${\hat{\theta }}_{F}$ represents the observers estimate of the vergence state $\theta_{F}$ and $\hat{h}$ their estimate of the half interocular separation h. With these estimates they can make a distance estimate ${\hat{D}}_{F}$. A final step relates this perceptual estimate to the report of this estimate as ${\hat{D}}_{R}$, e.g., by manual estimation or verbal report.

Equation 5 “inverts” the relationship between distance and vergence, geometrically allowing distance in the world to be estimated from vergence [9,18]. The strategy of inverting a generative model relating proximal cues to distal properties is termed “inverse optics” [19] and is central to contemporary models of perception, which formulate perception as a process of statistical inference [20–24]. Vergence is thought to primarily be of utility in judging near distances as its magnitude drops off rapidly with distance (Fig 2a).

Therefore, if a person can accurately estimate $\theta_{F}$ and h, and has an appropriate inverse generative model, they could accurately estimate distance, $D_{F}$. In the following we will use the “hat” symbol to represent the sensory system’s *estimate* of a property, be that distal or proximal. Thus, ${\hat{\theta }}_{F}$ represents the sensory system’s *estimate* of the *proximal* signal $\theta_{F}$, and ${\hat{D}}_{F}$ the sensory system’s *estimate* of the *distal* signal $D_{F}$. As such, if a person does not have accurate information about $\theta_{F}$ and/or h (${\hat{\theta }}_{F}\neq \theta_{F}$ and/or $\hat{h}\neq h$) they will misestimate distance, i.e., ${\hat{D}}_{F}\neq D_{F}$. Under these circumstances their perception would be said to be “non-veridical”, “biased” [25–28] or lacking “external accuracy” [29].

Given an internal estimate of distance ${\hat{D}}_{F}$, a further function $f_{R}$ transforms this estimate into a perceptual report $f_{R}({\hat{D}}_{F})={\hat{D}}_{R}$ (Fig 2b). If this is not an identity function a person will accurately estimate distance but incorrectly report that accurate estimate. This “response bias” could have both “perceptual” and “cognitive” components. An example of a “perceptual” component would be an incorrect mapping between the perceptual estimate and motor commands needed for a manual estimate. An example of a “cognitive” component would be a bias to report values toward the centre of the experimental stimulus range [30].

Whilst acknowledged to exist, response bias is a controversial, complicated topic typically assumed to be absent in models of statistical inference. The reason for this is that in the extreme it is currently an intractable perceptual and philosophical issue to establish if a person has correctly estimated a distal world property, but then incorrectly reported that correct estimate [31]. In this regard a “response bias” could effectively be an unprovable redescription of the estimation bias one is wanting to explain. What we are interested in here is modelling how observers might estimate distance from ocular convergence, not how they might estimate distance but then respond in a way inconsistent with their estimate.

### Estimating distance from ocular convergence: Experimental evidence

Despite over 150 years of research, there is still active debate as to whether observers can accurately estimate absolute distance from vergence (see [16] for a comprehensive review). Within the literature, the primary ways to measure perceived distance from vergence have been (1) verbal distance estimates, (2) manual distance estimates, (3) relative distance judgements and (4) judgements about other properties such as depth, motion or shape. Verbal distance estimates tend to be used less frequently and there is evidence that they can be more variable across observers (e.g., [32]), so we will primarily describe other response modes here as they are reflective of the literature.

In an early study, Swenson [33] had observers manually estimate the distance of a disc light source, the angular size and intensity of which were kept constant, and found highly accurate judgements within the 25–40 cm range. Gogel and Tietz [34] investigated the perception of further distances with an illusory motion parallax task (where incorrect distance estimation would result in a stationary light appearing to move either with or against lateral head movements) and concluded that observers overestimate near distances and underestimate far. They described this as being consistent with observers’ estimates of distance contracting toward an intermediate default value, a phenomenon termed the ‘specific distance tendency’ [35].

Later studies using manual distance estimates found largely accurate estimates in near visual space (less than approximately 60 cm) and suggested that any residual misestimation of distance from vergence could be due to a cognitive ‘contraction bias’, whereby people report perceptual estimates contracted to the centre of an available experimental range [9,18,30,36,37]. This is an example where it is suggested that distance might be accurately estimated but inaccurately reported. A similar process has been suggested to distort the results of studies seeking to estimate the relative importance of binocular cues in the control of grasping [38].

In a widely cited study, Viguier et al. [10] used behavioural measures and eye tracking to examine distance perception from vergence. Behavioural measures included manual distance matching, manual half- and double- distance setting and verbal report. All measures showed progressive underestimation of distance as physical distance increased. Given the additional steps in half and double distance setting we will focus on manual distance matching.

Here, when asking observers to manually set the distance of a point light cursor to match that of another previously seen target (leaving 5 seconds between presentations to eliminate any disparity cue between the cursor and target), Viguier et al. found that observers accurately estimated near distances, but progressively underestimated further distances, despite them correctly fixated the very same targets (as shown through measuring the physical orientation of the eyes).

All of the above studies used physical light sources, avoiding the conflicting cues that are typically present when using computer generated stimuli [39]. However, the issue of conflicting cues is still relevant. To “isolate” the vergence cue many studies hold cues such as retinal size and object luminance constant. For example, in Viguier et al. [10] the target had a fixed retinal size and luminance, whereas the matched point light cursor’s retinal size and luminance varied consistently with vergence-specified distance. Cue conflicts could therefore influence measured distance misestimation from vergence. This is an issue that affects virtually all studies of sensory cues (see [40] for a detailed exposition of problems inherent in isolating sensory cues).

Recently Linton [16] has suggested that all published studies claiming to show distance estimation from vergence are in fact the result of other uncontrolled cues to distance. Aiming to experimentally isolate vergence fully, Linton found that observers were insensitive to slow changes in vergence, and distance estimates did not track vergence when it was changed in this way. Puzzlingly, many observers made consistently non-random distance estimates suggesting that they were using some source of distance information or strategy and not simply guessing on each trial. Linton (p. 3187) acknowledges that “there are several alternative interpretations of these results that we cannot conclusively reject” but considered these all less plausible than observers not being able to judge distance from vergence.

### Indirectly inferring distance perception from ocular convergence: Experimental evidence

**Fig 3:**
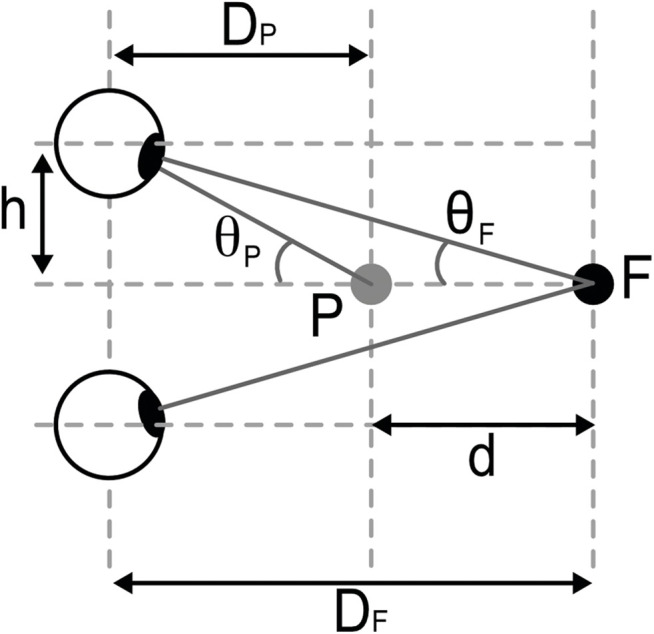
Diagram showing the geometry of estimating depth from retinal disparity. An observer fixates a point F which is at a distance $D_{F}$ directly in front of the observer in the median plane (this produces the half vergence angle $\theta_{F}$). A second point, P, on the median plane is placed at $D_{P}=D_{F}-d$ (which produces the half vergence angle $\theta_{P}$). This produces a half retinal disparity of η, which is equal to $\theta_{P}-\theta_{F}$.

In addition to having observers make direct distance estimates, tasks where depth and shape are estimated from retinal images have been widely used to infer vergence specified object distance and thus indirectly assess the accuracy of distance estimation from vergence (e.g., [41,42]). Consider an observer fixating at a distance $D_{F}$ in the median plane, with another point on the median plane is placed at $D_{P}=D_{F}-d$ (Fig 3) and the observer is asked to perceptually judge the depth d.

The half retinal disparity produced by this viewing geometry is given by


$$\eta ={\text{tan}}^{-\text{1}}\left(\frac{dh}{-dD_{F}+D_{F}^{\text{2}}+h^{\text{2}}}\right)$$ (6)

Rearranging Equation 6, we can see that one way in which an observer could estimate d is to scale retinal disparity by an estimate of $D_{F}$ provided by vergence together with knowledge of their interocular distance.


$$d=\frac{\left(D_{F}^{\text{2}}+h^{\text{2}}\right)\text{tan}(\eta )}{h+D_{F}\text{tan}(\eta )}$$ (7)

This process is termed ‘disparity scaling’, as the same disparity can be produced by infinitely many depths and distances, so the measured disparity signal needs to be “scaled” in order for depth to be estimated [8].

Due to this geometric relationship, investigating the perception of depth from disparity, where vergence is the sole or primary distance cue, has been used to infer information about the accuracy of distance estimation from vergence. For example, if an observer makes a depth estimate, $\hat{d}$, given an object of depth d at a distance $D_{F}$, where vergence is the only distance cue, we can infer the vergence specified distance, ${\hat{D}}_{F}$, that they used to scale retinal disparity. This “scaling distance” [41,42] is given by:


$${\hat{D}}_{F}=\frac{\text{1}}{\text{2}}\left(d+\sqrt{d^{\text{2}}-\text{4}h^{\text{2}}+\text{4}dh\;\text{cot}(\eta )}\right)$$ (8)

One of the most influential studies in this area is that of Johnston [41]. In this study, observers judged whether disparity-defined horizontally orientated elliptical hemi-cylinders, viewed at a range of distances, had a circular cross section. It was found that for a hemi-cylinder to have a perceived circular cross-section it had to be physically ‘squashed’ in depth extent at near distances (approximately <80 cm) and physically ‘stretched’ in depth at far distances (approximately >80 cm). This pattern of results has been replicated in numerous studies with different types of stimuli, either viewed on a computer monitor [25–27,42–48], via a virtual reality headset [49,50], or with real-world physical stimuli [39,51].

These results are consistent with a progressive underestimation of distance as physical distance increases, but with an additional overestimation of near distances. Similar experiments have shown that vergence is lawfully combined with information from vertical disparity (a retinal cue to binocular viewing geometry) when estimating depth [43,52]. Here, with each cue in isolation the inferred scaling distance was an overestimate at near distances and a progressive underestimate as distance increased, but with an improvement in scaling when both cues varied consistently [43].

It is difficult to determine whether the pattern of results of depth, size and shape judgement tasks can be used to directly infer vergence specified distance. This inference would be based upon assuming that scaled retinal disparity is the sole cue to the distal property being estimated (e.g., depth/shape/size) and that vergence is the sole cue providing the distance estimate used to scale retinal disparity. These are significant assumptions given the difficulty of isolating sensory cues [40]. Additionally vergence responses can be driven independent of perceived depth [53] and depth can be perceived from diplopic images independent of vergence state [54]. Therefore, in what follows, we focus on direct distance estimation from vergence, rather than distance estimates inferred indirectly.

### Summary of the present paper

We present a Bayesian model of how an observer might estimate the distal property of absolute distance from the proximal ocular vergence signal. Our focus is not on resolving the longstanding experimental debate as to whether observers can accurately estimate distance from vergence. Rather, our aim is to rigorously examine how an observer might do this based upon the proximal signal available to the sensory system. We find that the progressive underestimation of distance with increasing physical distance observed in experimental studies can be predicted to arise from observers trying to estimate the most likely distance in the world to have produced a measured but uncertain vergence signal. The amount of vergence uncertainty needed to account for the misestimation of distance is directly comparable to that which has been experimentally measured [55,56].

## Methods and results

### Probability of world distance from ocular convergence

Sensory signals are inherently stochastic [57]. As a result, for a given physically identical proximal cue observers can make repeated sensory estimates that differ from one another. One of the most widely used methods to model perceptual estimation from stochastic (noisy) signals is Bayesian inference. In this framework perception is seen as a process of inverting the generative function relating measured noisy proximal sensory cues to distal world properties. To do this, current sensory data is combined with prior knowledge that the observer holds about the probability of distal properties of the world. The estimate made from this probabilistic information is determined by the resulting posterior probability density function and the cost associated with making perceptual errors [20,23,58].

We adopt the Bayesian framework here to examine how observers might estimate absolute distance from vergence. Our approach is consistent with other studies that have examined how binocular viewing geometry and estimation from noisy sensory signals [59–64] contribute to our perception of distal world properties. All derivations and simulations reported in the paper were produced using Mathematica 14.1 in conjunction with the Mathstatica plugin (version 2.73) [65] run on a M3 Pro MacBook Pro (macOS 14.6.1). Where possible, computations were spread over all cores using the parallel computing functionality in Mathematica and Mathstatica.

We first define a probability density function $P\left(\hat{\theta }\right)$ that specifies the likelihood of a given measured vergence state $\hat{\theta }$ with fixation on a point F at a distance $D_{F}$ on the median plane (Fig 1b). For a given value of $D_{F}$ there is a specific probability of observing any given vergence state $\hat{\theta }$. Let D be the domain of $D_{F}$, i.e., all possible distances in the world ($D:\;\text{0}<D_{F}<\infty$) and let θ be the domain of $\hat{\theta }$, i.e., all possible vergence states given these distances ($\theta :\;\text{0}<\hat{\theta }<\frac{\pi }{\text{2}}$).

As is standard, we assume that an observer has an unbiased measurement of $\theta_{F}$ corrupted by zero-mean Gaussian noise with a standard deviation of σ.


$$\hat{\theta }\sim N\left(\theta_{F},\sigma^{\text{2}}\right)$$ (9)

Given the domain D, this defines a truncated normal likelihood function, $p\left(\hat{\theta }|\theta_{F}\right),$ where $\theta_{F}$ is the true half-vergence angle corresponding to fixation on distance $D_{F}$ and erf the standard error function $erf(z)=\frac{\text{2}}{\sqrt{\pi }}\int_{\text{0}}^{z}e^{-t^{\text{2}}}\text{d}t$.


$$p\left(\hat{\theta }|\theta_{F}\right)=\frac{\sqrt{\frac{\text{2}}{\pi }}}{\sigma \left(erf\left(\frac{\theta_{F}}{\sqrt{\text{2}\sigma }}\right)-erf\left(\frac{\theta_{F}-\frac{\pi }{\text{2}}}{\sqrt{\text{2}\sigma }}\right)\right)}e^{-\frac{{\left(\theta_{F}-\hat{\theta }\right)}^{\text{2}}}{\text{2}\sigma^{\text{2}}}}$$ (10)

Here, This likelihood function is defined as a function of vergence angle $\theta_{F}$, not of distance $D_{F}$, which is the distance property of the world being estimated. As such we need to reformulate $p\left(\hat{\theta }|\theta_{F}\right)$ in terms of distance rather than vergence angle (Equation 5). To do this we recognise that for a random variable X, distributed according to $f_{X}(x)$, if Y=g(X) is a differentiable monotonic transformation then Y is also a random variable [66].


$$f_{Y}(y)=f_{X}\left(g^{-\text{1}}(y)\right)\left|\frac{d}{dy}g^{-\text{1}}(y)\right|$$ (11)

Here $f_{X}(x)$ is the truncated normal probability density function representing the likelihood of observing a vergence angle $\hat{\theta }$ given that the true vergence angle is $x=\theta_{F}$. $f_{Y}(y)$ is transformed probability density function representing the likelihood of observing a vergence angle $\hat{\theta }$ given that the true fixation distance is $y=D_{F}$.

The inverse of the transform we wish to apply is


$$g^{-\text{1}}(y)=\theta_{F}={\text{tan}}^{-\text{1}}\left(\frac{h}{D_{F}}\right)$$ (12)

Whilst the tangent function is periodic over π, it is bijective and monotonic over the vergence domain D. We can therefore use a *change of variables* to perform a closed form transform between probability density functions.

Differentiating [12] with respect to $D_{F}$ gives


$$\frac{d}{dy}g^{-\text{1}}(y)=-\frac{h}{h^{\text{2}}+D_{F}^{\text{2}}}$$ (13)

Substituting [10] and [13] into [11] gives


$$p\left(\hat{\theta }|D_{F}\right)=\frac{\sqrt{\frac{\text{2}}{\pi }}}{\sigma \left(erf\left(\frac{\theta_{F}}{\sqrt{\text{2}\sigma }}\right)-erf\left(\frac{\theta_{F}-\frac{\pi }{\text{2}}}{\sqrt{\text{2}\sigma }}\right)\right)}e^{-\frac{{\left(\theta_{F}-\hat{\theta }\right)}^{\text{2}}}{\text{2}\sigma^{\text{2}}}}.\left|\frac{h}{h^{\text{2}}+D_{F}^{\text{2}}}\right|$$ (14)

With y∈R, D>0 and h>0 this gives the transformed likelihood function


$$p\left(\hat{\theta }|D_{F}\right)=\frac{h\sqrt{\frac{\text{2}}{\pi }}}{\left(h^{\text{2}}+D^{\text{2}}\right)\sigma \left(erf\left(\frac{\theta_{F}}{\sqrt{\text{2}\sigma }}\right)-erf\left(\frac{\theta_{F}-\frac{\pi }{\text{2}}}{\sqrt{\text{2}\sigma }}\right)\right)}e^{-\frac{{\left(\theta_{F}-\hat{\theta }\right)}^{\text{2}}}{\text{2}\sigma^{\text{2}}}}$$ (15)

Here $\theta_{F}={\text{tan}}^{-\text{1}}\left(\frac{h}{D_{F}}\right)$ is the true vergence angle for the fixation distance $D_{F}$, and $\hat{\theta }={\text{tan}}^{-\text{1}}\left(\frac{h}{D_{i}}\right)$, i.e., the internal vergence angle measurement corresponding to a possible distance value $D_{i}$.

Therefore, for a given value of noise, σ, in the proximal vergence signal, the likelihood function $p\left(\hat{\theta }|D_{F}\right)$ represents current sensory information from vergence that can be used to estimate distance in the world.

In the Bayesian framework this likelihood function is combined with a prior over the distance ($p(D_{F})$) using Bayes rule to obtain a posterior distribution of possible distances from vergence states.


$$p\left(D_{F}|\hat{\theta }\right)\propto p\left(\hat{\theta }|D_{F}\right)\cdot p\left(D_{F}\right)$$ (16)

The prior represents the *a priori* belief of encountering any possible distance in the world independent of current sensory information. For now, following numerous other studies [2,26,39,67–72], we make the assumption that the prior for distance is flat, or so broad as to be noninformative compared to the likelihood function. However, we examine this assumption below.

With this assumption, the posterior probability distribution has the same form as the likelihood function:


$$p\left(D_{F}|\hat{\theta }\right)=p\left(\hat{\theta }|D_{F}\right)=\frac{h\sqrt{\frac{\text{2}}{\pi }}}{\left(h^{\text{2}}+D^{\text{2}}\right)\sigma \left(erf\left(\frac{\theta_{F}}{\sqrt{\text{2}\sigma }}\right)-erf\left(\frac{\theta_{F}-\frac{\pi }{\text{2}}}{\sqrt{\text{2}\sigma }}\right)\right)}e^{-\frac{{\left(\theta_{F}-\hat{\theta }\right)}^{\text{2}}}{\text{2}\sigma^{\text{2}}}}$$ (17)

**Fig 4:**
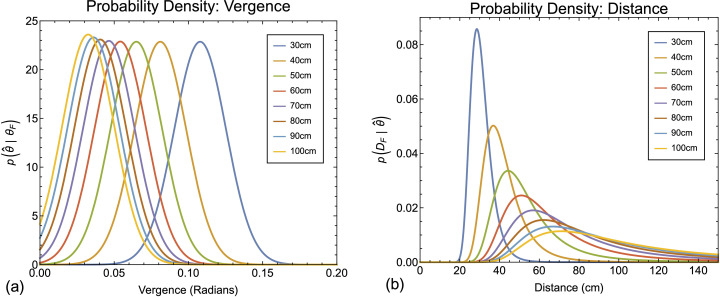
Probability density functions for vergence and distance. (a) Gaussian probability density functions for vergence (Equation 10) for distances between 30 cm and 100 cm. In all cases the sigma of the truncated Gaussian is 1 degree. (b) Corresponding probability density functions for distance from vergence (Equation 17). These are plotted for fixation distances at 10 cm intervals between 30 cm (roughly the nearest distance a person can comfortably fixate) and 140 cm (a distance at which more than 90% of the vergence range is used [8]).

By inverting the generative model between distance and vergence we can express likelihood functions for vergence (Fig 4a) as corresponding posterior probability distributions for distance from vergence (Fig 4b). In Fig 4, for illustrative purposes, we use a value of 1 degree for σ [55,56]. We consider a range of values below.

The non-linear relationship between vergence and distance transforms the truncated Gaussian likelihood functions for vergence estimates from vergence $p\left(\hat{\theta }|\theta_{F}\right)$ into positively skewed likelihood functions for vergence estimates from distance $p\left(\hat{\theta }|D_{F}\right)$ and therefore (assuming a flat prior) distance estimates from vergence $p\left(D_{F}|\hat{\theta }\right)$. This transformation has this form because any given value of noise δ in the vergence signal $\theta_{F}$ corresponds to larger “overestimation” versus “underestimation” errors in distance [18]. That is


$$\left(\frac{h}{\text{tan}\left(\theta_{F}-\delta \right)}\right)-D_{F}>D_{F}-\left(\frac{h}{\text{tan}\left(\theta_{F}+\delta \right)}\right)$$ (18)

### Making a perceptual estimate

The estimate of distance, $\hat{D}$, made from the posterior distributions shown in Fig 4b depends on the loss function defining the costs associated with misestimating the distal property of interest. The loss is itself a random variable $L\left({\hat{D}}_{F}|D_{F}\right)$, so the goal of the decision process is to minimise the expected loss associated with a given decision, commonly termed risk, $R_{{\hat{D}}_{F}}\left(D_{F}\right)$ [65].


$$R_{{\hat{D}}_{F}}\left(D_{F}\right)=E\left(L\left({\hat{D}}_{F}|D_{F}\right)\right)$$ (19)

The loss function is critical because it determines the perceptual estimate that is made from the posterior. This estimate is often termed an “optimal” estimate (e.g., Ernst and Banks [67]) due to it minimising the risk associated with a perceptual decision. However, the structure of the loss function is typically unknown and there are infinitely many possibilities. As a result, in most applications of Bayesian modelling the structure of the loss function is assumed (although see [73] for an attempt at an experimental estimate).

Here, to avoid assuming a loss function, but also to make our analysis tractable, following [74], we examine three of the most commonly used loss functions in Bayesian modelling. These are the quadratic loss:


$$L\left({\hat{D}}_{F}|D_{F}\right)={\left(D-D_{F}\right)}^{\text{2}}$$ (20)

The absolute error loss:


$$L\left({\hat{D}}_{F}|D_{F}\right)=\left|D-D_{F}\right|$$ (21)

And the zero-one loss:


$$\begin{array}{c} L\left({\hat{D}}_{F}|D_{F}\right)=\left\{ \;\;\;\begin{array}{c} \text{0}\;if\;\left|D-D_{F}\right|\;\leq \varepsilon \\ \text{1}\;if\;\left|D-D_{F}\right|\;>\varepsilon \end{array}\right.\; \end{array}$$ (22)

For the zero-one loss, ε is the value of error ‘acceptable’ to the observer, which defines an interval within which all estimates accrue equal loss. Therefore, when ε>0 there are a range of perceptual estimates which would accrue equal loss. When ε→0, the loss function approaches a Dirac Delta function, and the decision rule results in a single estimate. With a uniform prior and ε=0 this is termed Maximum Likelihood Estimation (MLE) observers would be trying to estimate the most likely world property to have caused the measured noisy sensory cue [67].

For the quadratic loss function the estimate minimising risk is the mean of the posterior, for the absolute loss function it is the median and for the zero-one loss function it is the peak [74]. However, in many applications of Bayesian modelling the likelihood, prior and posterior distributions are assumed to be Gaussian over the domain of the units used for estimation [2,39,67–72,75,76]. In this case all three decision rules discussed above result in the same ‘optimal’ estimate being made. However, for many cues, the likelihood, prior and posterior will not be Gaussian, and these decision rules will provide different estimates. This makes assuming a loss function problematic.

Here, the peaks of $p\left(D_{F}|\hat{\theta }\right)$ were estimated using Mathematica’s *FindMaximum* function with the Conjugate Gradient method. It was not possible to find a simple closed form solution for the mean (expectation) of the $p\left(D_{F}|\hat{\theta }\right)$, so we calculated this by numerical integration over the range 10 cm (estimated minimum possible vergence angle) to 6m (estimated upper limit on the utility of vergence as a cue to distance). One might argue that a larger domain should be used for integration, as observers are able to use vergence and stereoscopic cues at much larger distances then is commonly thought [77]. For the present paper this does not matter as increasing the domain of integration simply results in a greater progressive overestimation of distance (due to the tail of the skewed posterior distribution). Conversely, decreasing the domain decreases the overestimation but never results in underestimation.

To determine the median, we convert $p\left(D_{F}|\hat{\theta }\right)$ into a cumulative density function:


$$CDF\left(p\left(D_{F}|\hat{\theta }\right)\right)=\frac{erf\left(\frac{-\text{2}\theta_{F}+\pi }{\text{2}\sqrt{\text{2}}\sigma }\right)+erf\left(\frac{\theta_{F}-\hat{\theta }}{\sqrt{\text{2}}\sigma }\right)}{erf\left(\frac{\theta_{F}}{\sqrt{\text{2}}\sigma }\right)-erf\left(\frac{\theta_{F-}\frac{\pi }{\text{2}}}{\sqrt{\text{2}}\sigma }\right)}$$ (23)

**Fig 5:**
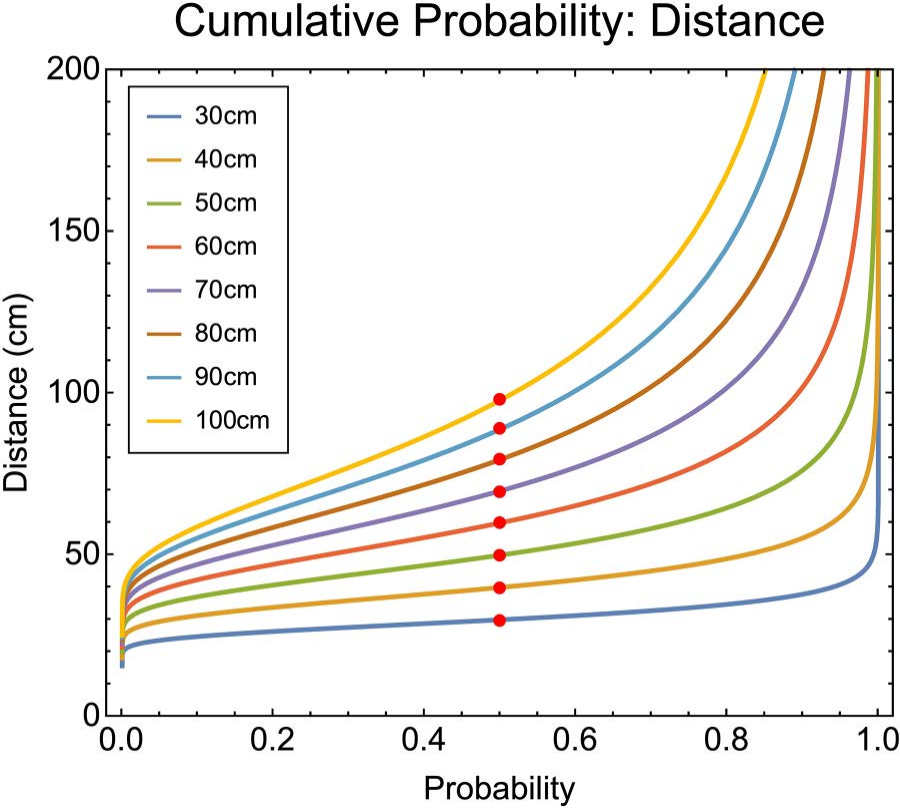
Inverse cumulative density functions for distance from vergence (corresponding probability density functions for distance shown in Fig 4b). The red points show the median of the functions. We show the inverse CDF’s, rather than the CDF’s, for ease of visualisation.

We then solve this function at the 0.5 point. For a given $p\left(D_{F}|\hat{\theta }\right)$ this represents the distance value at which 50% of the probability density has accumulated (Fig 5).

**Fig 6:**
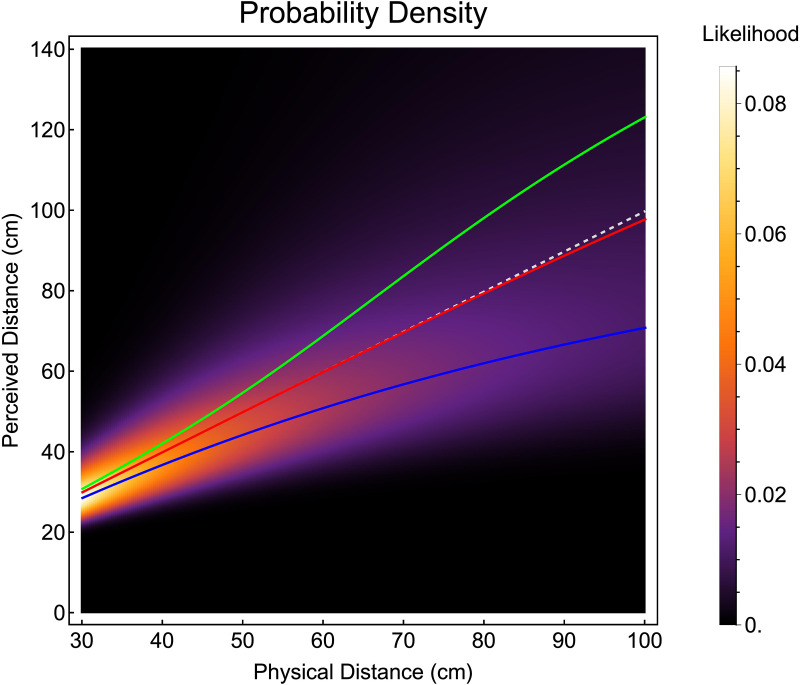
Probability density functions for perceived distance (vertical pixels), overlaid with distance estimates made by choosing the peak (blue line), expectation (green line) or median (red line) of the distributions. The diagonal dashed grey line represents veridical performance, i.e., accurate estimates of distance.

The image in Fig 6 plots the likelihood of perceived distance across a range of physical distances. Each column of pixels is a posterior probability density function as shown in Fig 4b. Overlaid on the image are the peak (blue line), expectation (green line) and median (red line) of the distributions. The three cost functions result in distinct predictions regarding the estimation of distance from vergence. The expectation predicts a progressive overestimation of distance as vergence-specified distance increases, which is the opposite pattern to that found in the literature. The median predicts virtually no perceptual bias at all, again, inconsistent with the experimental literature. By contrast, the peak results in a progressive underestimation of distance consistent with the experimental literature.

**Fig 7:**
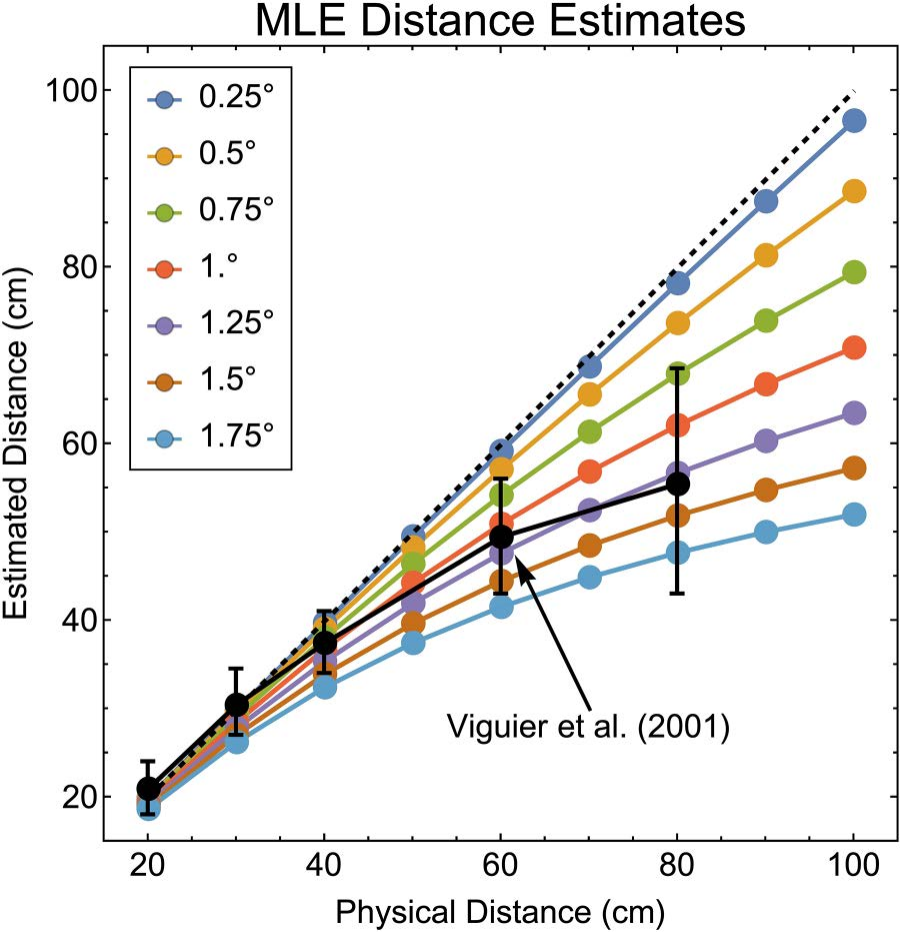
Maximum likelihood estimates of perceived distance (uniform steps of 10 cm between 20 cm and 100 cm) from ocular convergence for a range of noise values (uniform steps of 0.25° between 0.25° and 1.75°). The diagonal dashed grey line represents veridical performance, i.e., accurate estimates of distance. The black datapoints are manual distance estimates from ocular convergence from Viguier et al. (2001) as described above (error bars show ±SD). Note that the lines in the graph represent point estimates from the posterior distributions, not estimates over the course of an experiment. We compare the Viguier et al. data to direct predictions from response distributions below (see main text).

Given that the zero-one loss function was the only one to predict an underestimation of distance consistent with that found in the experimental literature, we further examined its properties to see whether it could quantitively, as well as qualitatively, predict distance underestimation from vergence. Fig 7 shows how the progressive underestimation of distance by choosing the peak lawfully varies for a range of noise levels in the vergence signal. As vergence noise increases, the underestimation of further distances also increases, with near distances being estimated most accurately. Overlaid for comparison is the data from Viguier et al. [10].

Mon-Williams and Tresilian [18] recognised the asymmetric effect of noise in distance from vergence (Equation 18) and the effect that this would have for average distance estimates (they did not consider the nature of the posterior distribution), and proposed that “(i)n an attempt to compensate for this, the system could incorporate an underestimation bias for more distant targets” (p. 177). Here we show that with a different cost function this “necessarily speculative” (p. 177) suggestion is not needed. We next examine whether response distributions predicted by this model are consistent with data from the experimental literature.

### Response distributions for distance from ocular convergence

The posterior probability density functions shown in Figs 4b and 6 represent the information available to the observer at a given instant in time, for example the point at which a perceptual estimate is made on a trial of an experiment. However, across trials the posterior probability density function will not be identical due to the stochastic nature of sensory noise. As such, despite often being assumed, posterior probability density functions do not necessarily show the same distribution as perceptual estimates across trials in an experiment (for discussion see [23]).

**Fig 8:**
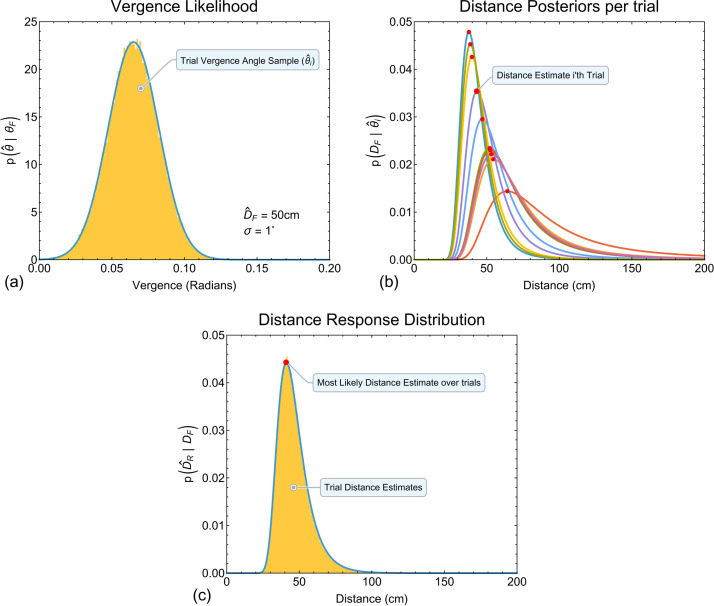
Illustrative example of how response distributions were calculated. (a) For a given distance and vergence noise level (here $D_{F}=\text{5}\text{0}cm$ and $\sigma_{i}=\text{1}\circ$), for each simulated experimental trial i, a random sample ${\hat{\theta }}_{i}$ (across trials collectively illustrated by the orange histogram was drawn from the likelihood function for vergence $p\left(\hat{\theta }|\theta_{F}\right)$ (blue distribution curve). Each sample ${\hat{\theta }}_{i}$ represents the observer’s measured vergence state on that trial. (b) This produces a unique trial-by-trial posterior $p\left(D_{F}|{\hat{\theta }}_{i}\right)$, here shown for ten examples. The peaks of the posteriors (red dots) were taken as the observer’s distance estimates. (c) The response distribution (orange histogram) is the distribution of posterior peaks across trials. The blue distribution curve is the best fitting parametric distribution (see main text for details), the peak of which was taken as the most likely distance estimate an observer would make across trials for that specific distance and vergence noise level.

For a given physical distance $D_{F}$ and vergence noise level $\sigma_{i}$, to estimate response distributions $p\left({\hat{D}}_{R}|D_{F}\right)$ across trials in a hypothetical experiment, the following procedure was followed (Fig 8). On each trial i a sensory observation ${\hat{\theta }}_{i}$ was drawn from the distribution $p\left(\hat{\theta }|\theta_{F}\right)$ (Fig 8a). This represents the fact that for a constant value of $D_{F}$ an observer’s measured vergence angle will be different across trials due to sensory noise. This produces a unique trial-by-trial posterior distribution of distance from vergence $p\left(D_{F}|{\hat{\theta }}_{i}\right)$ (Fig 8b). The peak of the posterior was taken as the observer’s distance estimate on that trial. We simulated 100000 trials to build response distributions for a given physical distance and vergence noise level (Fig 8c).

**Fig 9:**
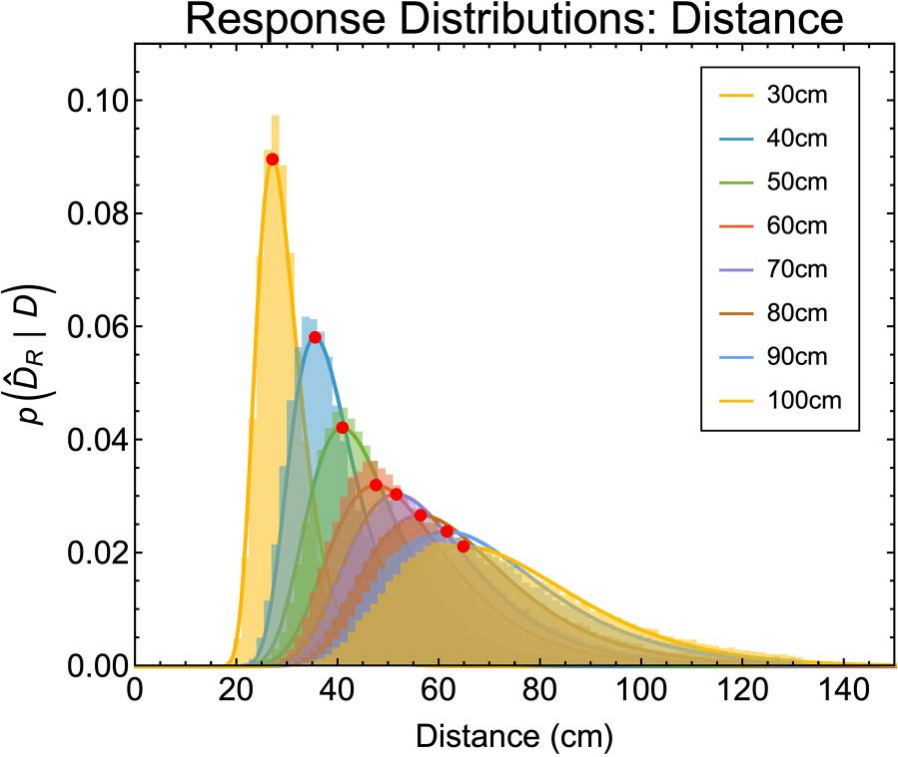
Shows example response distribution histograms for distance estimates across trials for a range of distances and a vergence noise value of 1°. Solid lines show the best fitting parametric distribution. Red points show the peaks of these parametric distributions.

Example response distributions for all distances with a vergence noise value of 1*°* are shown in Fig 9. Response distributions are skewed in a similar fashion to the per-trial posterior distributions. Overlaid on these histograms are best fitting parametric distributions found using Mathematica’s *FindDistribution* function (this function uses the Bayesian information criterion together with priors over possible distribution types to select both the best distribution and the best parameters for that distribution). These functions represent a description of the data, not a computational model, thus, the best fitting parametric distribution (and its parameters) can differ across simulated data (identical conclusions are made throughout if we instead fit smooth kernel distributions). The peaks of the fitted parametric distributions (red dots in Fig 9) were taken as the most likely distance estimate an observer would give *across trials* for a given distance and vergence noise value.

**Fig 10:**
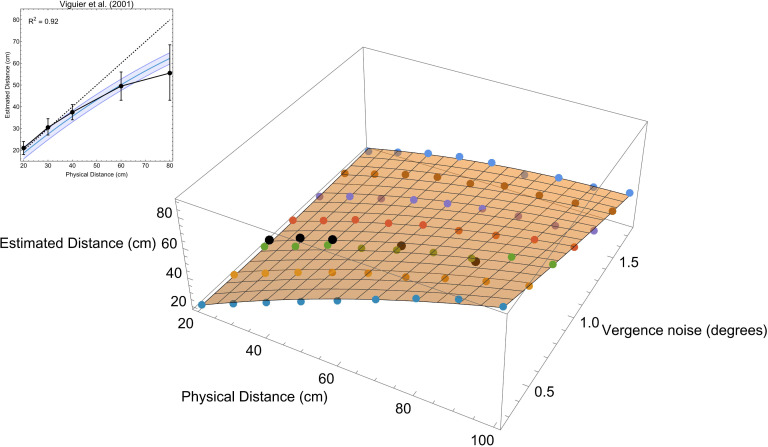
The main image shows peaks of response distributions for estimating distance from a noisy vergence signal (purple points) with the best fitting quadratic surface (semi-opaque orange surface). The black points show the Viguier et al. [10] (error bars show ±SD). The data from Viguier et al. are positioned on the vergence noise axis such that the distance between the data and the fitted surface are minimised (minimum of the sum of squared differences). Inset shows the Viguier et al. data and the slice through the quadratic surface at this point. The fit to the data is excellent $R^{\text{2}}=\text{0}.\text{9}\text{2}$. The shaded region in the inset shows 95% confidence intervals for single predictions.

Peaks of the response distributions for a range of vergence noise values (uniform steps of 0.25*°* between 0.25*°* and 1.75*°*) and distances (uniform steps of 10 cm between 20 cm and 100 cm) are shown in Fig 10 (main plot). Given these data we can infer the amount of noise in the vergence signal that would be required to account for the distance underestimation observed in Viguier et al. [10]. To do this the data were fit by least squares with a quadratic surface (x, y, $x^{\text{2}}$, $y^{\text{2}}$, and xy terms). A second-order polynomial was chosen not as a model of the data but as a simple way of describing the data. The fit of the surface to the data was excellent, $R^{\text{2}}=\text{0}.\text{9}\text{9}$.

Next we minimised the sum of the squared differences between the Viguier et al. [10] data and the fitted surface using Mathematica’s *Minimize* function. This suggested that given our Bayesian model of distance estimation, a vergence noise of σ=0.79∘ would be needed to produce the experimentally observed distance misestimation in Viguier et al. [10]. This value is directly comparable with experimental estimates of the magnitude of vergence noise [55,56]. The minimised fit of the vergence model to the Viguier et al. data is shown inset in Fig 10. The fit captures the experimental data very well $R^{\text{2}}=\text{0}.\text{9}\text{2}$. Possibly the experimental data more rapidly shifts to distance underestimation compared to the model, however, this is difficult to definitively quantify given the error bounds around the experimental data (especially at larger distances).

### Bayesian priors for distance

Here we assumed that the prior for distance was flat or so broad as to be noninformative compared to the likelihood function, but we know that distances in the world are not uniformly distributed [78–80]. The choice of prior was made for two reasons. First, Bayesian models have been criticised for the proposition of priors to explain patterns of experimental data with little justification, i.e., the prior is just a redescription of the pattern in the data the experimenter wants to account for [81]. Second, whilst substantial progress has been made in directly measuring the statistical structure of the world (for an overview see the discussion section of [82]), there remain significant difficulties, rarely acknowledged, in interpreting measured frequency distributions of distal world properties as internal Bayesian priors.

A typical approach to measuring a distance prior is to place a scanner at positions in the environment and measure a frequency count of radial distance. The experimenter must make choices such as where in the environment to sample and normally positions the scanner at a minimum distance from objects so that they do not occlude the scanners full field of view. These choices affect the measured distributions. Scanners also have a minimum and maximum measurable distance, meaning that distances outside its range, by definition, cannot exist in the database. Minimum distances in current databases are around 2m (e.g., 3m in [78], 1m in [80], and approximately 2m in [83]).

We spend a significant amount of time interacting with objects within arm’s reach, so databases such as these do not contain most of the distances we interact with. This is particularly problematic for modelling cues such as vergence, whose utility drops off rapidly with distance (Fig 2a). Scanners also sample the scene uniformly across their field of view, which is not true for humans who sample the scene based upon factors such as interest, saliency and task [84–86].

A key tenet of Bayesian modelling and Bayesian statistics in general [74] is that priors, likelihoods and posteriors *do not* simply represent frequency distributions, rather they represent distributions of *belief*; in our case, our belief about distances in the world. This allows Bayesian models to be derived to reason about properties that cannot be measured directly, which is not possible from a frequentist perspective. This is also why we ensured that the choice of a truncated normal distribution for the noisy vergence signal, rather than a normal distribution, did not affect our results. Belief about physically impossible distances could exist for a true Bayesian observer.

A final critical assumption is that observers accurately estimate the values of the distal property in the database, otherwise the measured frequency distribution will not represent the internal perceptual prior. One could argue that we have access to a rich array of sensory cues, coupled with the ability to calibrate cues based on sensory feedback [87,88], thus distance should be accurately estimated. However, this is inconsistent with a wide body of literature showing that observers misestimate absolute distance (and most other distal properties) in both simulated and real environments, with rich availability of visual cues [89–93]. Furthermore, integration of sensory cues to make a perceptual estimate does not necessarily result in accurate calibration of those same cues [26,94].

### Internalisation of Bayesian priors

Given the above, we are not in the position to infer a distance prior in order to examine its effects on the perceptual estimation of distance from vergence. We can however examine the effect that biased perceptual estimation of distance from vergence could have on internalising a distance prior. To do this we simulated 200 semi-natural scenes in Matlab 2024b using 3D meshes of scanned natural objects (apple, pumpkin, sweetcorn, pomegranate, lemon, pinecone, nectarine, tomato, ginger, kiwi, garlic, carrot, sprout, and avocado) (see S2 Text). The high-resolution 3D meshes were loaded into Maltab using the gptoolbox [95].

**Fig 11:**
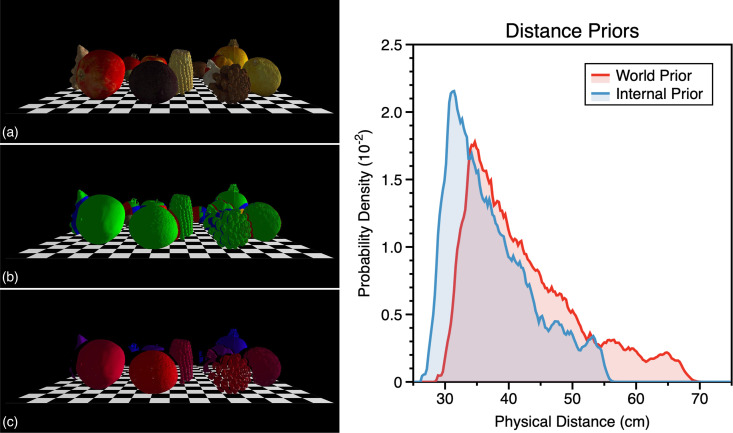
Estimating a distance prior. (a) Example scene created from scanned objects, the scene is rendered on a checkboard surface for visualisation, (b) same scene colour coded to show the portions of the objects viewable from each eye (green = both eyes, red = right eye only, blue = left eye only), (c) same scene now showing only the vertices viewable from both eyes, colour coded for radial distance from the cyclopean eye (red = near, blue = far), (d) distance priors calculated over all 200 scenes, red shows the distal prior present in the world, blue shows the prior which gets estimated from the vergence signal. Note: that in (a-c) the checkerboard surface is shown for ease of visualisation and was not part of the raytraced scenes. Additionally, the simulated viewpoint has been set to aid visualisation of the raytracing.

Meshes were randomly positioned on a 35 cm square planar surface area such that bounding circles around the objects, in the x-z plane, did not intersect (Fig 11). Meshes were positioned in the y dimension such that their minimum y coordinate touched the floor plane. Placement was sequential and if this resulted in all objects not being able to be placed in the scene without their bounding circles intersecting the scene was discarded. This process was repeated to create the 200 semi-natural scenes. In total there were just over 8 million mesh vertices per scene.

Each scene was then raytraced to determine the visibility of every mesh vertex to each eye. To do this we raytraced each scene as viewed by an observer with an interocular distance of 6.5 cm positioned at x = 0 cm, y = 8 cm and z = 60 cm relative to the centre of the 35 cm square planar area. Raytracing was performed using parallel processing with Embree (https://www.embree.org) accessed via the libigl C++ geometry processing library [96], called through the gptoolbox [95].

An example scene is shown in Fig 11a. This is shown again artificially coloured for visibility from each eye in Fig 11b and for radial distance to each vertex (from the cyclopean eye) viewable from both eyes in Fig 11c. Radial distances across scenes together constituted our world distance “prior” (Fig 11d). Note, that for the reasons stated, we are not claiming that this is a true estimate of the world prior for distance. Indeed, the structure of the prior is completely irrelevant for our reasoning.

Distances in the prior were then passed through the transform relating physical to perceived distance from vergence for the best fitting vergence noise level (Fig 10). Passing the world prior through this transform results in a number of interesting properties. Some far distances present in the physical scene are absent in the internalised prior and the probability of the remaining distances is contracted towards near distances. Additionally, some distances that do not exist in the world prior exist in the internalised prior. This highlights the critical importance of understanding how properties of the world are estimated when interpreting statistical priors derived directly from environmental measurements.

### At what stage(s) of processing do priors act?

Existing studies generally implement priors at the level of the distal property being modelled, for example, distance [78] or slant/tilt [83]. However, it is possible that a prior could act at an earlier stage of processing. In the case of distance estimation from ocular convergence, one possibility is that rather than, or in addition to, a prior for distance (the distal property being estimated) one could have a prior for the convergence state of the eyes (the measured proximal sensory signal). In some senses priors over proximal signals could be considered more realistic as the sensory system has direct access to the signal. This would remove assumptions related to the distal property being accuracy estimated from the proximal signal for it to be instantiated in a prior.

**Fig 12:**
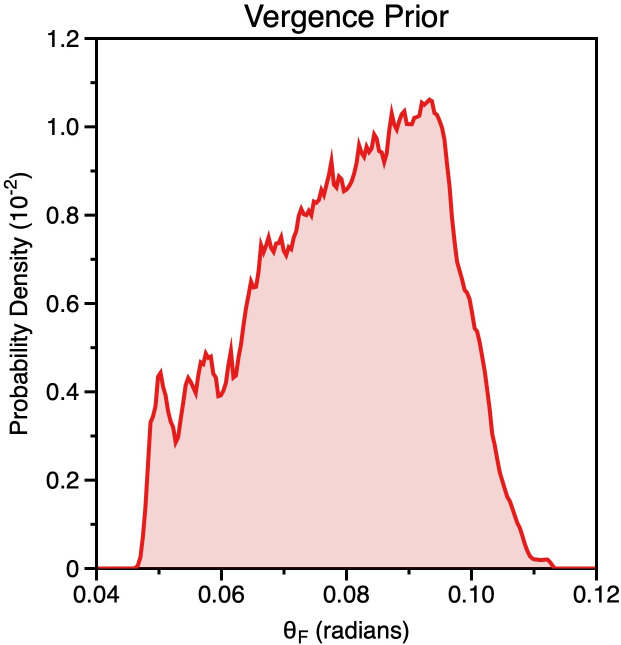
Vergence prior, calculated by passing the radial distances in the world prior in Fig 11b through the transform relating distance to ocular convergence (Equation 4) with an assumed interocular distance of 6.5 cm.

In Fig 12 we show the “vergence prior” that would be produced by an observer fixating the distances in the world prior shown in Fig 11. As is clear, the prior is not flat, as we have assumed for our modelling.

Indeed, we can use the same change of variables technique as above to derive the distribution of world distances $p\left(D_{F}\right)$ that would result in a flat vergence prior (S3 Text).


$$p\left(D_{F}\right)\;=\frac{h}{\left(-b+a)(h^{\text{2}}+D^{\text{2}}\right)}$$ (24)

**Fig 13:**
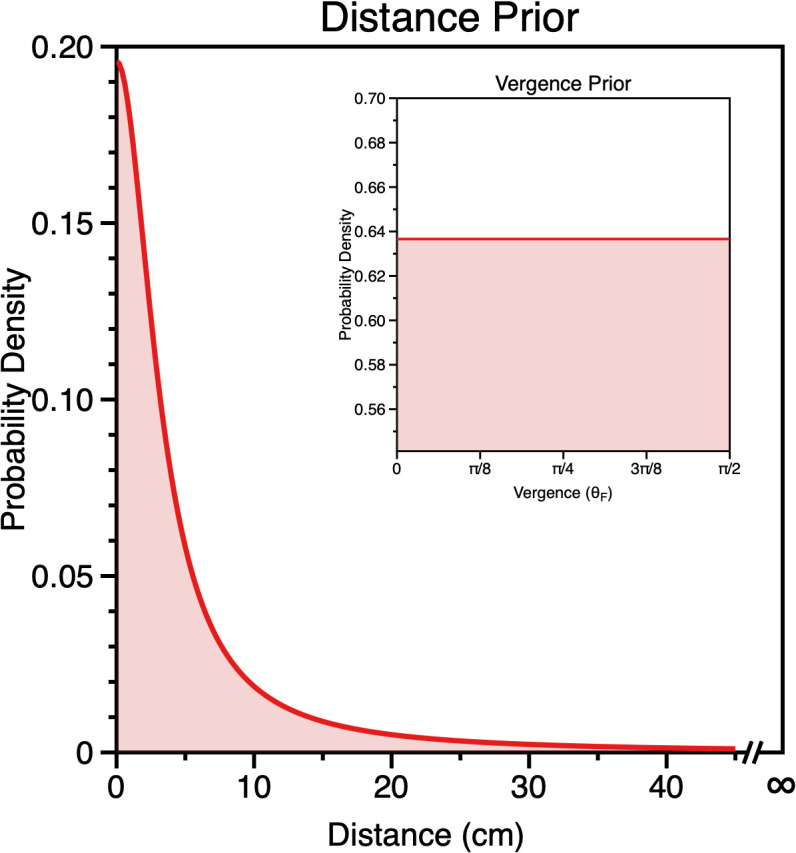
Distance prior which would be consistent with a flat vergence prior (inset). The domain of vergence is $\theta :\;\text{0}<\hat{\theta }<\frac{\pi }{\text{2}}$, as such the probability density function is a constant $\frac{\text{2}}{\pi }$. The corresponding domain for distance is $D:\;\text{0}<D_{F}<\infty$.

In Fig 13 we plot $p\left(D_{F}\right)$ with a=0, $b=\frac{Pi}{\text{2}}$ (i.e., the full vergence domain θ), and h=3.25cm (half the average interocular distance). As can been seen, the distance prior needed to produce a flat vergence likelihood has a greater probability of near distances compared to far, with a peak at 0. The preponderance of near distances would be consistent with far distances being less likely due to occlusion and perspective projection [97], so in this sense a flat vergence prior is consistent with far distances being less likely to be encountered. However, it is not clear that a peak is realistic or physically meaningful [86] or that the true world prior for distance would decrease in this way. This again highlights the problems inherent in interpreting priors (and using them to account for perceptual bias).

This leads to the question of how such a “vergence prior” could be instantiated. In darkness the eyes are known to adopt a tonic state (dark vergence) of approximately 90 cm to 1m [98]. This could be interpreted as a “prior” if it influenced distance estimation in the low light typical of experimental settings. It would likely have to operate after the stage at which vergence state (and its uncertainty) has been measured, as mis-convergence could cause diplopia. This is consistent with distance from vergence being underestimated despite correct physical convergence [10]. The control of convergence is also closely coupled to the accommodative system, which works in concert to maintain clear single binocular vision. The accommodative system has a tonic state (dark focus) around the same value as dark vergence.

If interpreted as a prior, the tonic state of the vergence and accommodation systems is not consistent with the progressive underestimation near distances. Instead, it would predict near distance overestimation as distance estimates would be pulled towards the centre of mass of the prior ~ 1m. Whilst we are not averse to considering priors operating at multiple processing levels, we are very wary of overinterpreting any prior for the reasons previously stated. We have therefore focused on examining how distance might be (mis)estimated from a noisy ocular convergence signal without resorting to a prior, which is normally the route taken to explain perceptual bias.

## Discussion

### Summary

We have presented a Bayesian model of the perception of distance from ocular convergence. For a vergence noise value consistent with that measured experimentally [55,56] this model can account for the progressive underestimation of distance, despite accurate convergence [10]. This is achieved by observers trying to estimate the most likely distance to have produced a measured noisy vergence signal. Helmholtz ([99] p.318) recognised the importance of the accuracy and precision of the vergence signal in making correct inferences about properties of the world when stating that:

*“Owing to the uncertainty of our judgements as to the degree of convergence of the eyes, we are liable to have illusions also about the forms of things in space as seen binocularly. The interpretation of the visual phenomena would be correct if the amount of convergence were different, but it is not correct of the convergence actually used.”*

It is not clear that our Bayesian model was exactly what Helmholtz had in mind in this quote, but it confirms that there are circumstances where the “degree of convergence” can be correct, but “uncertainty” in estimating this state can result in the misperception of absolute distance.

At all points we have aimed to be consistent with previous literature and be as transparent as possible in the modelling choices made. We are aware that Bayesian models offer a high degree of flexibility and can be criticised on this basis [81]. We have emphasised the critical importance of considering the nature of the transform function relating distal world properties to proximal sensory data, and the nature of the loss function chosen. Many examples of Bayesian modelling disregard the former and make assumptions about the latter. We have also highlighted the significant assumptions made in using Bayesian priors to account for perceptual bias, whether these priors are assumed, inferred or experimentally measured.

Our aim was not to experimentally resolve the debate around the experimental evidence of human use of vergence to estimate distance [3,10,16,43,55,100–103]. Rather, it was to rigorously analyse how the distal property of absolute distance might be estimated from the proximal vergence signal. Given the flexibility in scope of Bayesian models, we too made some modelling assumptions and simplifications, which we detailed. These include Gaussian noise in the vergence signal (here of a fixed value), an uninformative distance prior, and examination of three of the loss functions (despite their being infinitely many potentials). Future work could clearly examine the landscape of possible models further.

### Experimentally measuring vergence noise

We would also like to highlight the difficulty one faces in experimentally inferring the level of noise in the vergence signal. One could measure the physiological state of the eyes using an eye tracker, however the inferred value could be affected by many factors related to the eye tracker, e.g., recording method, onboard filtering and sampling rate, recording duration, task and the specific metrics derived from the data [104]. Physiological measurements also do not take into account any upstream processes beyond the physical orientation of the eyes, so will likely underestimate the noise in the vergence signal. Alternatively, one could get observers to make absolute distance estimates from vergence and based on the variability of these infer the level of noise in the vergence signal. This is also problematic as it is extremely difficult to truly isolate the vergence cue from other cues to distance [16,40].

Vergence noise has been measured psychophysically using a nonius line method, in which an observer judges the horizontal distance between two vertical lines, one presented to each eye with a vertical offset between the two (i.e., a relative judgement). When estimated using this technique, the standard deviation of vergence decreases with distance, from 9.5 arc min to 4.0 arc min between 40 cm and 100 cm [105] and 3.75 arc min at 210 cm (Chopin et al., 2016). These values are much smaller than those used in our model and decrease with distance. In contrast, Brenner and van Damme [55] estimated a standard deviation for vergence noise when used as a cue to distance of 50–60 arc min. This latter value, which reflects the uncertainty in our ability to access vergence information as an absolute cue to distance, is much closer to the value required in our model to account for perceptual biases.

While the reason for this discrepancy is not clear, it is an example of the ‘absolute disparity anomaly’ [106] that describes how our ability to access vergence or disparity information to make absolute distance judgements is much impaired in comparison to its use in relative judgments. Chopin et al. [106] argue that, while relative depth judgments are based on absolute disparity information, the latter is not directly accessible for absolute depth judgements, at least not with the same precision as they are used in relative judgements. As a result, absolute depth thresholds have been reported as anything between three [107,108] and thirty [109] times higher than relative depth.

Similarly, the uncertainty of distance judgements from vergence is a factor of around 10–20 times poorer than the level of vergence noise measured using a nonius task [105]. Brenner and van Damme [55] estimated the standard deviation for changes in distance from changes in vergence to be much lower (around 10 arc min) than for absolute judgements, in line with measures of vergence noise from a nonius task [55,105,106,110,111].

Variation in estimates will also be affected by numerous simple, but critical, aspects of the experimental procedure. For example, the time interval between presenting an object whose distance is to be estimated, and the object used to manually estimate that distance [10,55]. A time interval is needed to eliminate a disparity cue but introduces a memory component which will affect the inferred noise level. Tasks used to indirectly infer vergence specified distance suffer from the same problems and introduce numerous additional assumptions (discussed above).

As such, our focus has been on examining how an observer might, in principle, estimate distance from vergence within a normative Bayesian model, examining a range of noise values and common cost functions. We hope this provides guidance for experimentally testing models of distance estimation from vergence going forward and emphasises the importance of considering the generative function relating the measured proximal signal to distal world property being estimated.

## Supporting information

S1 Text Explanation of nodal point geometry. (DOCX)

S2 Text Description of how objects were 3D scanned. (DOCX)

S3 Text Description and equations for the flat vergence prior. (DOCX)
