# Supplementary material for: A Bayesian model of distance perception from ocular convergence
Source: PLoS Comput Biol. 2025 Oct 3;21(10):e1013506. doi: 10.1371/journal.pcbi.1013506 (PMC12513659; doi:10.1371/journal.pcbi.1013506)
Supplement: S1 Text — (DOCX) [file pcbi.1013506.s001.docx]

# S1: Nodal point geometry

For the purposes of the geometry described in the main paper we have defined the interocular distance to be the distance between the centre of rotation of each eye, and considered the eyes to be rigid, spheres which rotate about their centres. These are all standard conventions (14) but known to be a simplification of a more complicated anatomy. Firstly, the eyes are non-spherical objects which deform in geometry as they rotate, making any definition of the eyes geometry far from simple. We are unaware of any studies of the geometry of vergence and binocular vision which have taken this into account.

The assumption of the interocular distance being the distance between the centres of each eye is also a simplification of a more complex geometry. Optically, the interocular distance is more accurately defined as the Euclidean distance between the nodal points of each eye (S1A Fig). Thus, $h$ and $D_{F}$ directly depend on the convergence state of the eyes. If we define the distance between the nodal point of the eye and its centre to be $D_{NP}$. This means that in Equation 4 in the main text, $h$ needs to be corrected by subtracting ${\Delta h=D}_{NP}*sin(\theta_{F})$ and $D_{F}$ by subtracting ${\Delta D_{F}=D}_{NP}*cos(\theta_{F})$.


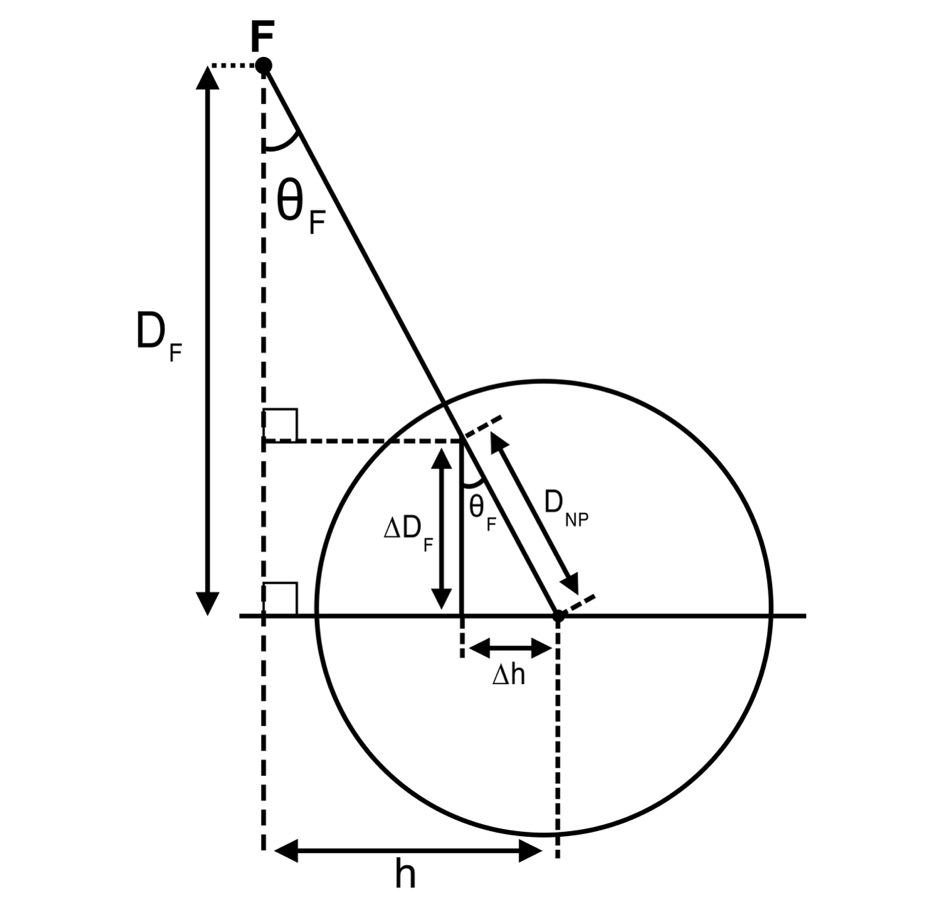


***S1A Fig****: Geometry of ocular convergence incorporating the eyes nodal point. For clarity of the geometry in the image we have greatly increased the scale of the eye in relation to the distance of fixation. This does not affect the mathematics of the geometry, but greatly helps with the visualisation of that geometry.*

If the average human eye has a radius of approximately 1.2cm and $D_{NP}$ is approximately 75% of this distance and considering a minimal comfortable convergence distance to be 30cm and a maximum distance in which vergence is considered a useful cue to be 600cm, S1B Fig plots $\Delta h$ and $\Delta D_{F}$. In terms of percentages of $h$ and $D_{F}$, these range between 0.15 and 2.98 percent. It is a completely open question as to whether the brain has “knowledge” of the eye’s geometry in terms of the interocular distance, nodal point and deformable structure. Therefore, we consider these assumptions in the main paper justified and consistent with previous research (14).


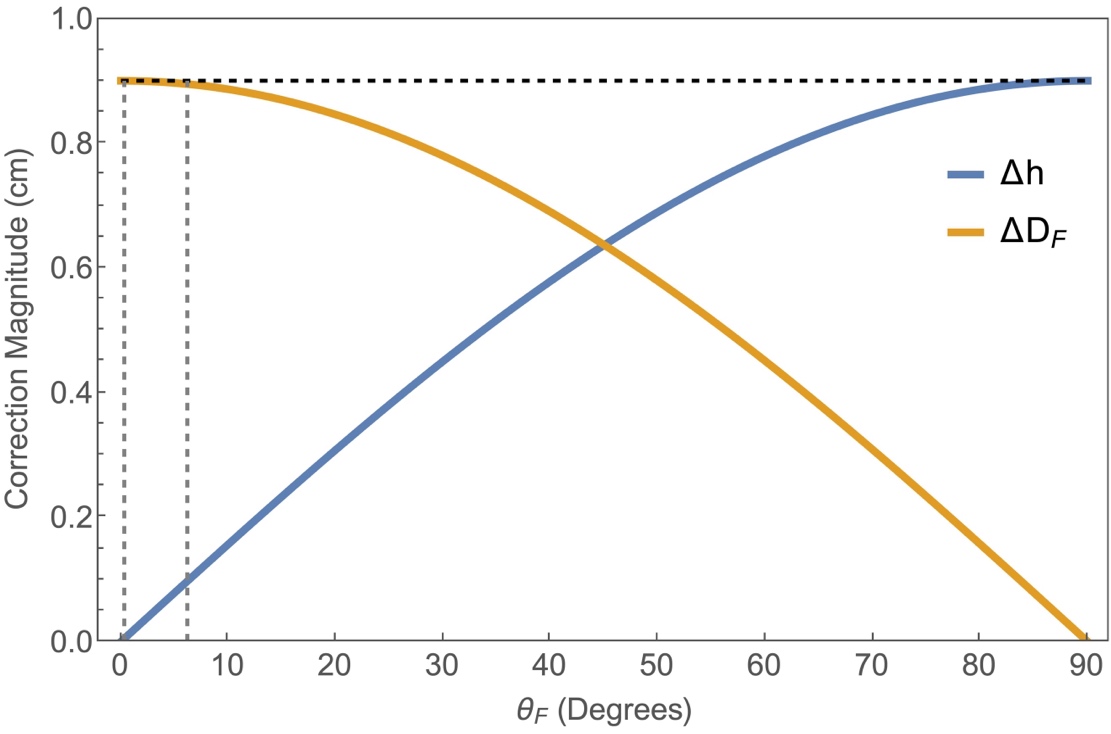


***S1B Fig****: Corrections in the geometry of vergence needed to account for the offset between the nodal point of the eye and the eyes centre of rotation. The graph shows the full angular range of 0 to 90 degrees. The vertical dashed lines show the vergence range equivalent to* $D_{F}=30-600cm$*. The horizontal dashed line shows* $D_{NP}$*.*
