# Supplementary material for: A Bayesian model of distance perception from ocular convergence
Source: PLoS Comput Biol. 2025 Oct 3;21(10):e1013506. doi: 10.1371/journal.pcbi.1013506 (PMC12513659; doi:10.1371/journal.pcbi.1013506)
Supplement: S2 Text — (DOCX) [file pcbi.1013506.s002.docx]

# S2: Object scanning

Objects were scanned using a NextEngine 3D Scanner and Multidrive turntable (settings: 360° rotation with eight divisions, two tilt settings of ±20°, 40k points/in^2^ and the “macro” distance range). Scans were edited in NextEngine Scan Studio HD: the turntable was removed with the ‘trim’ feature and the two tilted meshes were volume-merged with a resolution ratio 0.9, then fused with a 0.0025” tolerance, creating a water-tight mesh. Meshes were saved as .ply files in ASCII format and converted into Alias Wavefront Object (.obj) files via MeshLab (114).
