# Supplementary material for: A Bayesian model of distance perception from ocular convergence
Source: PLoS Comput Biol. 2025 Oct 3;21(10):e1013506. doi: 10.1371/journal.pcbi.1013506 (PMC12513659; doi:10.1371/journal.pcbi.1013506)
Supplement: S3 Text — (DOCX) [file pcbi.1013506.s003.docx]

# S3: Flat vergence prior

We can infer the distribution of distance in the world that would be consistent with a flat vergence prior, as assumed in our modelling, using the same change of variables approach detailed in the main text.

A flat vergence prior is given by the uniform distribution

$$p\left( \theta_{F} \right)=\frac{1}{b-a}$$

(S1)

Here, $0<a<b<{Pi}/2$, due to the domain of vergence $\boldsymbol{\theta}$.

Applying the same change of variables transform, with the same constraints, the equivalent of Equation 14 in the main text given by

$$p\left( D_{F} \right)=\frac{1}{b-a}.\left| \frac{h}{h^{2}+D_{F}^{2}} \right|$$

(S2)

This gives a distance prior of

$$p\left( D_{F} \right)=\frac{h}{(-a+b)(h^{2}+D_{F}^{2})}$$

(S3)
